# Passive Wireless LC Proximity Sensor Based on LTCC Technology

**DOI:** 10.3390/s19051110

**Published:** 2019-03-05

**Authors:** Mingsheng Ma, Yi Wang, Feng Liu, Faqiang Zhang, Zhifu Liu, Yongxiang Li

**Affiliations:** 1 CAS Key Laboratory of Inorganic Functional Materials and Devices, Shanghai Institute of Ceramics, Chinese Academy of Sciences, 1295 Dingxi Road, Shanghai 200050, China; mamingsheng@mail.sic.ac.cn (M.M.); liuf@mail.sic.ac.cn (F.L.); zhangfq@mail.sic.ac.cn (F.Z.); 2 Department of Electronic, Electrical and Systems Engineering, University of Birmingham, Birmingham B15 2TT, UK; 3 School of Engineering, RMIT University, Melbourne VIC 3001, Australia; yongxiang.li@rmit.edu.au

**Keywords:** proximity sensor, eddy current, LC resonance, LTCC technology

## Abstract

In this work, we report a passive wireless eddy current proximity sensor based on inductive-capacitive (LC) resonance using a low temperature co-fired ceramic (LTCC) technology. The operation principle of the LC proximity sensor to the metal targets was comprehensively discussed through electromagnetic simulation and circuit model. Copper and aluminum were selected as the metal target materials for the measurements. Circular copper plates with different diameters and thickness were used to investigate the influence of the surface area and thickness of the target on the sensitivity. The decreases of the sensitivity with the decrease of the surface area and thickness were observed. The LC proximity sensor showed a high sensitivity of 11.2 MHz/mm for the proximity distance of 1–3 mm, and large detection range up to 10 mm. The developed LC proximity sensor is promising for passive wireless metal detections and proximity measurements under harsh environments.

## 1. Introduction

Proximity sensors can detect the presence of nearby objects without any physical contact, and they have been widely used in the industry for displacement measurements, machine vibration monitoring, non-destructive testing, etc. [1]. Depending on the sensing targets and application scenarios, various proximity sensors based on different operation principles have been developed. Optical, capacitive, and eddy current sensors are the mostly used types of non-contact proximity sensors. Optical sensors can provide the best accuracy, but they are usually bulky and quite expensive due to their complex structure [2]. Capacitive sensors can achieve extremely high resolution and stability. However, the measurement results would be significantly affected if there were dirt, dust, oil or any dielectric materials between the capacitive sensor and target [2]. Since magnetic fields are insensitive to environmental contaminants, eddy current proximity sensor based on the magnetic coupling between the inductive coil of the sensor and the metal target is usually the first choice for most online measurement applications [3]. It is notable that the previously reported eddy current proximity sensors were commonly driven by external excitation currents, and normally required a complex signal and data process system, as with many active sensors [4]. However, in harsh environments and limited accessible space, it is highly desirable to use a passive wireless eddy current proximity sensing technique.

Inductive-capacitive (LC) resonance is one of the most promising methods to achieve passive wireless eddy current proximity detection for metal targets. Passive wireless LC sensors have been used for measuring pressure, temperature, humidity, chemical gas and biological parameters [5]. It is worth mentioning that most of the current passive wireless LC sensors use polymer substrates that are low cost in manufacturing and suitable for flexible and wearable electronics [6]. However, in harsh environments such as elevated temperatures, high degree of radiation or caustic chemical exposure, LC sensors based on more durable ceramics are better options. Low temperature co-fired ceramic (LTCC) with the multilayer co-fireable structure allows integration capacitors, inductors, and various sensing elements, and thus is considered an ideal approach to make LC sensors [7,8]. Passive wireless LC pressure, temperature and gas sensors based on LTCC technology have been successfully developed [9,10,11]. Eddy current proximity sensors based on LTCC have been reported [12,13,14]. Nevertheless, the previous work mainly focused on improving the sensor performance through multilayer coil design or integrating driving electronics with planar coil into multilayer LTCC modules. The studies on LTCC based passive wireless LC proximity sensors are still rare.

In this work, a passive wireless LC proximity sensor has been prototyped by the LTCC technology. The effects of the target materials, surface area, and thickness on the sensitivity were investigated through electromagnetic simulations and measurements. The developed LC proximity sensor achieved a high sensitivity of 11.2 MHz/mm for the proximity distance of 1–3 mm, and large sensing range up to 10 mm.

## 2. Sensor Design and Fabrication

**Figure 1:**
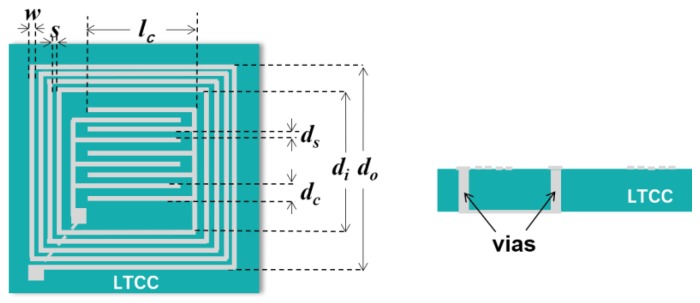
Schematic diagram of the inductive-capacitive (LC) proximity sensor based on low temperature co-fired ceramic (LTCC).

The schematic diagram of the LC proximity sensor is shown in Figure 1. A planar square spiral inductor (*L_s_*) and an interdigital capacitor (*C_s_*) were integrated on the LTCC substrate. They were interconnected through vias in the LTCC substrate to form a resonant circuit. The resonant frequency, *f*_0_, is related to *L_s_* and *C_s_* values as [15,16]:

(1) $$f_{0}=\frac{1}{2\pi \sqrt{L_{s}C_{s}}}$$

*L_s_* and *C_s_* depend on the structural dimensions of the circuit according to Reference [16]:

(2) $$L_{s}=1.39\times 10^{-6}\left(d_{0}+d_{i}\right)N_{i}^{5/3}\log\left(4\frac{d_{0}+d_{i}}{d_{0}-d_{i}}\right)$$

(3) $$C_{s}=l_{c}(N_{c}-1)\varepsilon_{0}\frac{1+\varepsilon_{r}K\left[{\left(1-{\left(d_{s}/d_{c}\right)}^{2}\right)}^{1/2}\right]}{2K\left(d_{s}/d_{c}\right)}$$

where *N_i_* is the turn number of the inductor, *d*_0_ and *d_i_* are the outer and inner widths of the inductor, respectively. *l_c_* is the length of the interdigital capacitor fingers. *N_c_* is the number of fingers in each terminal of the interdigital capacitor. *ε*_0_ is the free space permittivity. *ε_r_* is the dielectric constant of the LTCC substrate material. *d_c_* is the total spacing between two adjacent fingers. *d_s_* is the spacing between two adjacent fingers that is not covered by the conductor, and *K* is the complete elliptical integral of the first kind. The parameters for the designed LC proximity sensor are given in Table 1. 

The LC proximity sensor was fabricated using the LTCC process. The LTCC substrate material (SICCAS-K5F3) with a dielectric constant of *ε_r_* = 6.2 and dielectric loss tangent of tanδ = 0.001 (@10 GHz) was produced in-house at Shanghai Institute of Ceramics, Chinese Academy of Sciences (SICCAS). The thickness of a single green tape is about 50 μm. Details about the LTCC green tape preparation can be found in our previous paper [17]. Silver paste (Dupont, 6142D) was used for the metallization and deposited on the punched LTCC green tapes by screen-printing (P-200A, KEKO). The printed green tapes with eight layers were laminated at 70 °C under a pressure of 48 MPa for 10 min in an isostatic pressing chamber (LT08001, PTC). The punched vias were used for connecting the inductor and capacitor to form a closed resonant circuit. The vias were filled with silver paste and de-bindered at a heating rate of 1.5 °C/min from room temperature to 450 °C for 2 h. After organic burnout, the green body of the LC proximity sensor was sintered at a temperature of 850 °C for 10 min with a heating rate of 10 °C/min. The firing shrinkage of LTCC green tapes along X-Y direction is 16.7%. The firing shrinkage of LTCC green tapes along Z direction after lamination is 15.5%. The size of the LC proximity sensor after sintering was about 17.5 mm × 17.5 mm × 0.3 mm. 

## 3. Working Principle

**Figure 2:**
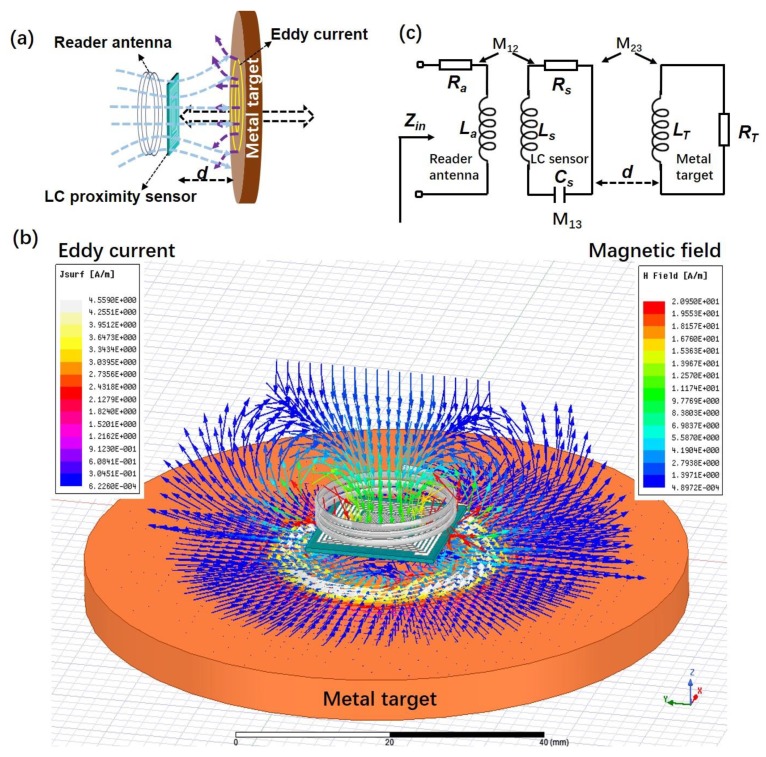
(**a**) Schematic diagram of the LC proximity sensor for metal target. (**b**) Electromagnetic simulation on the distribution of the eddy current and magnetic field. (**c**) Equivalent circuit of the proximity sensing system.

Figure 2a shows the schematic diagram of the work principle of LC proximity sensor for the metal target. The magnetic field is generated by the inductive coupling between the reader antenna and the LC proximity sensor. The magnetic flux can be supposed to cross over the metal target vertically when the metal target gets close to the sensor. Then, eddy currents are generated in the metal target according to Faraday’s law of induction. The eddy currents in turn produce an opposing magnetic field and result in the decrease of the inductance in the LC sensor. According to Equation (1), the change of the sensor resonant frequency, resulted from the change of inductance, can be detected wirelessly by measuring the impedance of an external coupled antenna as a function of frequency. For better understanding, the 3D model was created, as shown in Figure 2b. The distribution of eddy current and magnetic field vector was simulated by HFSS software. The induced eddy currents in the metal target can be observed, and their effect on the magnetic flux density of the LC proximity sensor coupled with the reader antenna was used for sensing the proximity of the target. 

The equivalent circuit of inductive coupling in the LC proximity sensor system is shown in Figure 2c. *R_a_* and *L_a_* represent the resistance and inductance of the reader antenna. *L_s_*, *C_s_* and *R_s_* refer to the inductance, capacitance, and resistance of the LC proximity sensor, respectively. The metal target is considered as an RL circuit [3]. *L_T_* and *R_T_* are the inductance and resistance of the metal target. *d* is the distance between the LC proximity sensor and metal target. *M*_12_, *M*_23_, and *M*_13_ are the mutual inductance between the reader antenna and sensor, the sensor and metal target, the reader antenna and metal target, respectively. The impedance (*Z_a_*) of the reader antenna, the impedances of the LC proximity sensor (*Z_S_*) and metal target (*Z_T_*) can be described using the Equations (4)–(6).

(4) $$Z_{a}=R_{a}+j\omega L_{a}$$

(5) $$Z_{s}=R_{s}+j\omega L_{s}+\frac{1}{j\omega C_{s}}$$

(6) $$Z_{T}=R_{T}+j\omega L_{T}$$

The mutual inductance can be described using Equations (7)–(9):

(7) $$M_{12}=k_{12}\sqrt{L_{a}L_{s}}$$

(8) $$M_{23}=k_{23}\sqrt{L_{s}L_{T}}$$

(9) $$M_{13}=k_{13}\sqrt{L_{a}L_{T}}$$

where *k* is the coupling coefficient [18]. Since the distance between the reader antenna and sensor is fixed in practical measurements, *M*_12_ is considered constant. However, the mutual inductance *M*_23_ decreases significantly with the increasing distance between the sensor coil and the target. *M*_13_ is ignored because it is much smaller than *M*_12_ and *M*_23_. For the whole proximity sensing system, the equivalent input impedance, inductance, and resistance from the reader antenna can be described as followed [19]:

(10) $$Z_{in}=R_{in}+j\omega L_{in}$$

(11) $$L_{in}=L_{a}+\frac{M_{12}^{2}}{M_{23}^{2}}L_{T}$$

(12) $$R_{in}=R_{a}+\frac{M_{12}^{2}}{M_{23}^{2}}R_{T}$$

Combing Equations (8) and (11), it can be found that *L_in_* is proportional to 1/$k_{23}^{2}$. As the coupling coefficient *k* increases with decreasing distance *d*, *L_in_* would decrease with decreasing *d*. From the above analysis, it can be seen that the variations of sensing distance *d* can be reflected by the measured impedance of the reader antenna.

## 4. Testing

**Figure 3:**
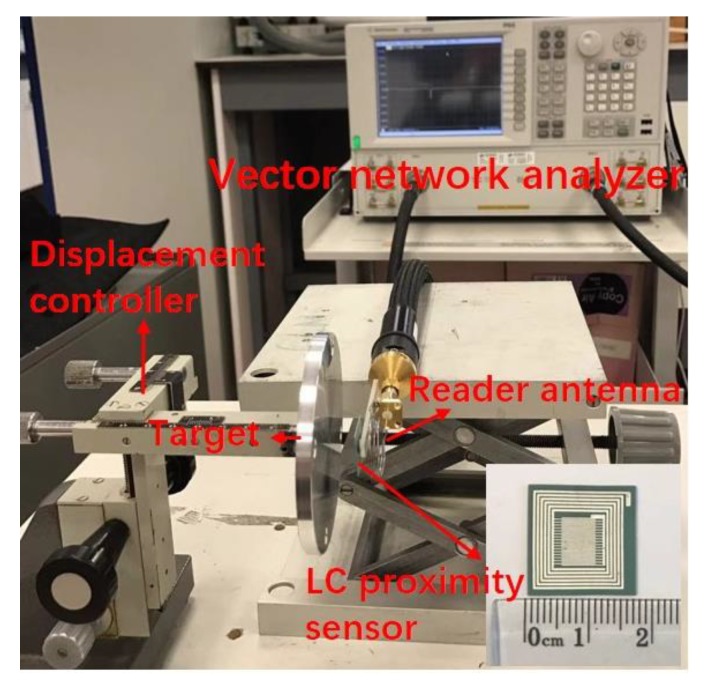
Measurement setup for the LC proximity sensor.

Figure 3 shows the photograph of the measurement setup for the LC proximity sensor. The metal target plate was fixed to a displacement controller. The distance between the sensor and the metal target was changed from 1 mm to 15 mm, and the test data was recorded once every 1 mm. Copper and aluminum were selected as the metal target material. Several circular copper plates with different diameters and thicknesses were used to investigate the influence of the surface area and thickness of the metal target on the sensor sensitivity. Information about the metal targets used in this work was given in Table 2. A wound wire reader antenna was connected to a vector network analyzer (E8362C, Agilent) and placed in parallel with the LC proximity sensor at a fixed separation of 5 mm. The radio wave reflected from the LC proximity sensor was collected by the reader antenna and was measured using this vector network analyzer. The reflection coefficient (*S*_11_) and corresponding resonance frequency, *f*_0_, were recorded. A calibration procedure was done by using a conventional one-port calibration method before the measurements.

## 5. Results and Discussion

**Figure 4:**
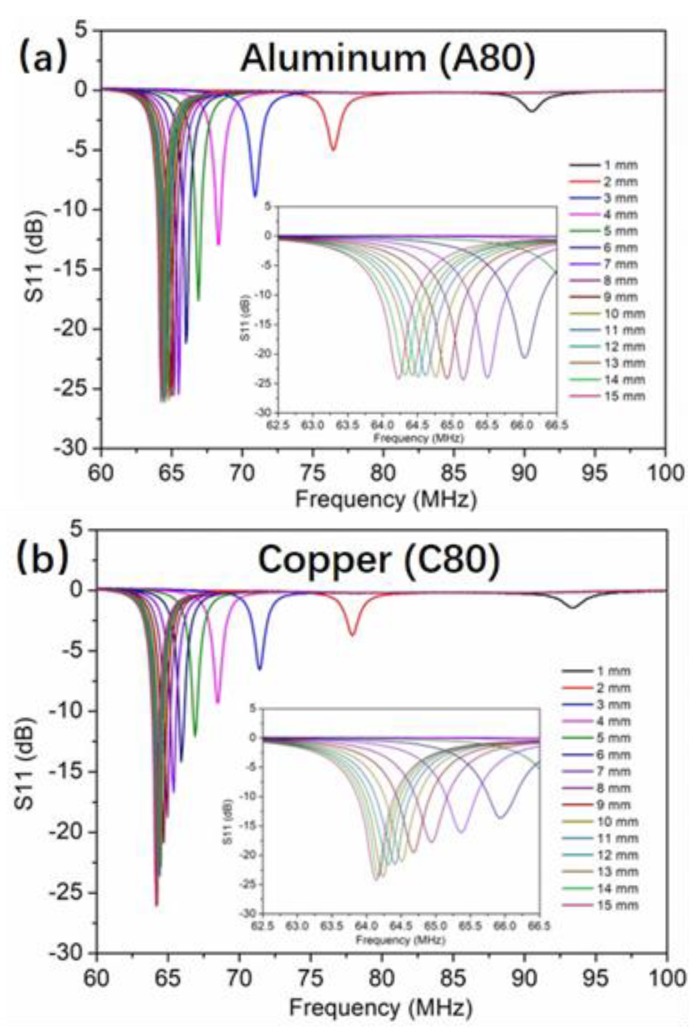
Response of the LC proximity sensor for the target of (**a**) A80 and (**b**) C80 at different testing distances.

Figure 4 shows the measured frequency response of the LC proximity sensor for the target of A80 and C80 of the same dimension at the sensing distance from 1 mm to 15 mm at a step of 1 mm. For both targets, the resonance frequency of the LC proximity sensor decreases with the increase of the sensing distance, while the magnitude of the resonance increases. According to Reference (11), the equivalent inductance of the proximity sensing system increases with the increase distance *d*, thus the resonance frequency reduces according to Reference (1). The magnitude of the resonance is mainly related to the quality factor (*Q* value) of the sensor. This can be described as [19]:

(13) $$Q=Q\left(d\right)=\frac{\omega L\left(d\right)}{R\left(d\right)}$$

where ω is the angular frequency. It can be seen that *Q* value depends on the sensing distance *d*, since both *L* and *R* are functions of the sensing distance *d*, according to the Equations (11) and (12). From the perceptive of energy, the *Q* value is proportional to the ratio of the stored energy to the total power losses within the component [20]. The longer the distance is, the smaller the eddy current loss is, and therefore the higher the *Q* value is. This results in the magnitude of the resonance increasing with the sensing distance. In addition, it can be observed that the magnitude of the resonance from the copper target is lower than the aluminum target at the same tested distance. This can be better appreciated by comparing the insets of Figure 4a,b. The reason is that the lower resistivity of the copper target causes a higher eddy current [21], and the *Q* value for the copper target was lowered as a result.

**Figure 5:**
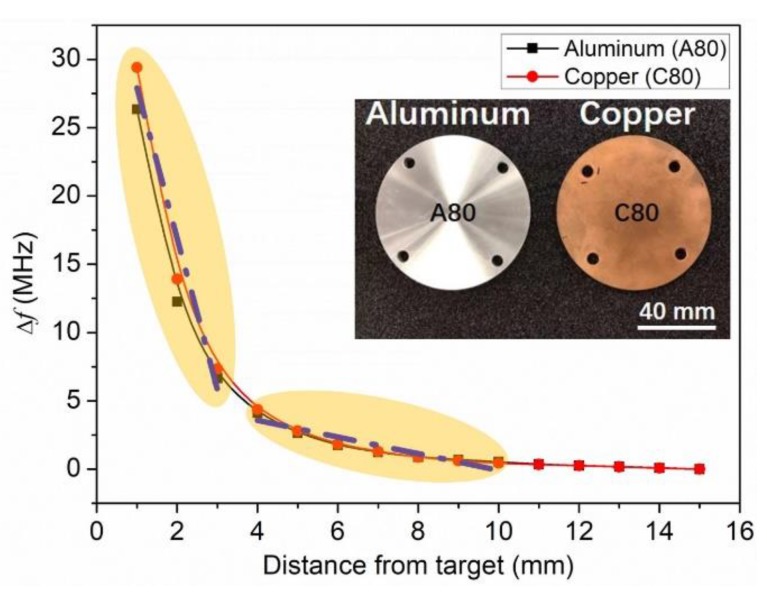
Frequency shift of the LC proximity sensor as a function of distance from the target for A80 and C80.

Since the frequency shift is more reliable than the *Q* value variation in the RF measurements, the difference (Δ*f*) between the original resonance frequency of the sensor and the resonance at different test distances was extracted as the sensing signal for the distance measurement, as shown in Figure 5. Compared with the aluminum target, the copper target causes slightly larger Δ*f*, and therefore has a higher sensitivity. Again, it can be attributed to the lower resistivity of the copper. There are two response sensitivity regions for both targets, as marked out in Figure 5. One is for the distance of 1–3 mm. By linear fitting, this region shows a high sensitivity of 11.2 MHz/mm, which is good for the accurate measurement in the micrometer scale. The other one is for the distance of 4–10 mm. By linear fitting, the sensitivity is about 0.6 MHz/mm, which is suitable for the long distance detection. It is worth mentioning that the sensing distance of the previously reported LTCC eddy current sensors is usually in millimeters ranges [12,13]. In Reference [13], they are able to measure up to 20 mm proximity distance of target, and the best sensitivity of presented inductive sensor was measured for distances up to 10 mm, repeatability measurement was verified for target distance from 4 to 7 mm. In this work, when the distance is larger than 10 mm, the signal resolution is near its limit. The nonlinear response of the LC proximity sensor over the whole sensing distance is due to the 1/*d*^3^ relationship of the inductor coils coupling coefficient *k* [22].

**Figure 6:**
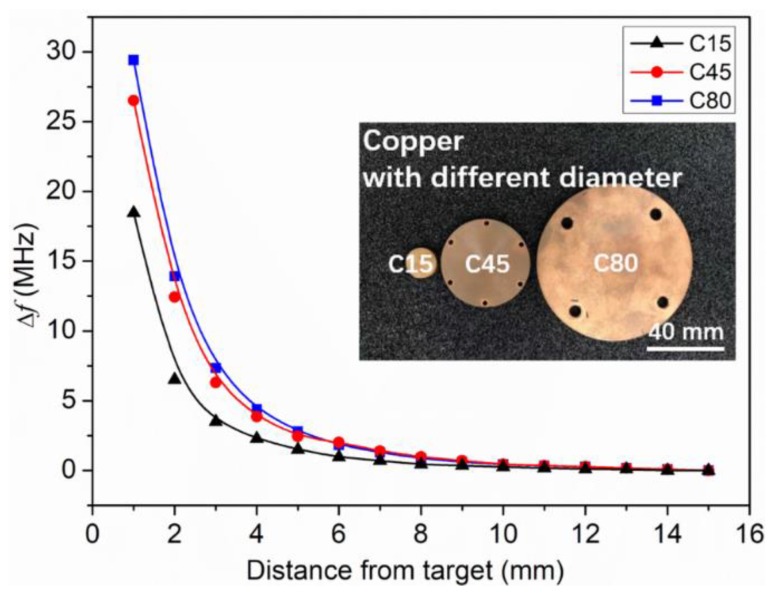
Frequency shift of the LC proximity sensor in dependence of the distance from the three targets of C15, C45, and C80.

**Figure 7:**
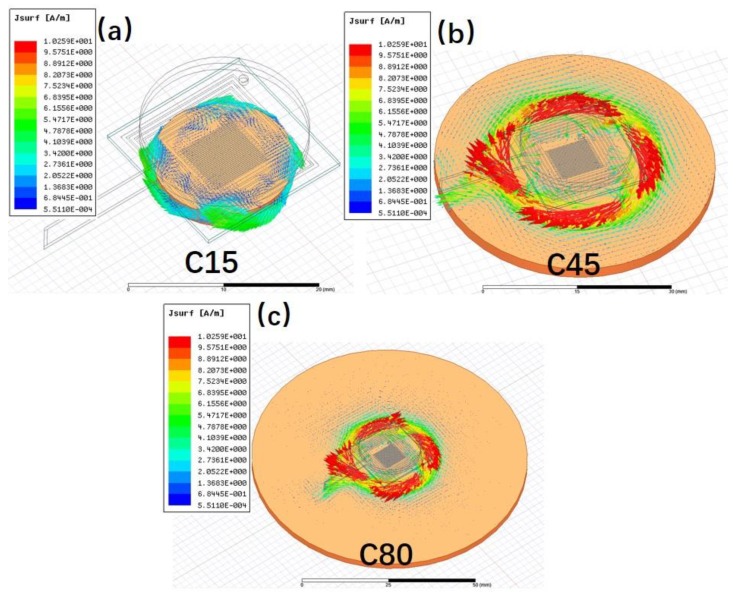
Simulated eddy current distribution for the target of (**a**) C15, (**b**) C45, and (**c**) C80.

The surface area of the metal target is considered as another important influencing factor to the eddy current. Three copper plates with the same thickness but different diameters of 15, 45, and 80 mm (namely C15, C45 and C80, respectively) were used to investigate their effects on the sensor performance. Figure 6 shows the frequency shift of the LC proximity sensor in dependence of distance from the target for the three samples. It can be seen that the Δ*f* increases with the increase of the surface area. This can be attributed to the increased influence on the sensor inductance from a larger target, which also means enhanced sensitivity of the sensor [22]. Compared with C45 and C80 targets, the Δ*f* of the C15 target is the smallest. This is because the surface area of C15 is smaller than that of the LC proximity sensor, and truncates the eddy current, as the simulated results shown in Figure 7. In this case, the change of sensor inductance induced by the C15 target would be reduced. This implies that the surface area of the metal target should be bigger than that of the LC proximity sensor for the better practical measurements.

**Figure 8:**
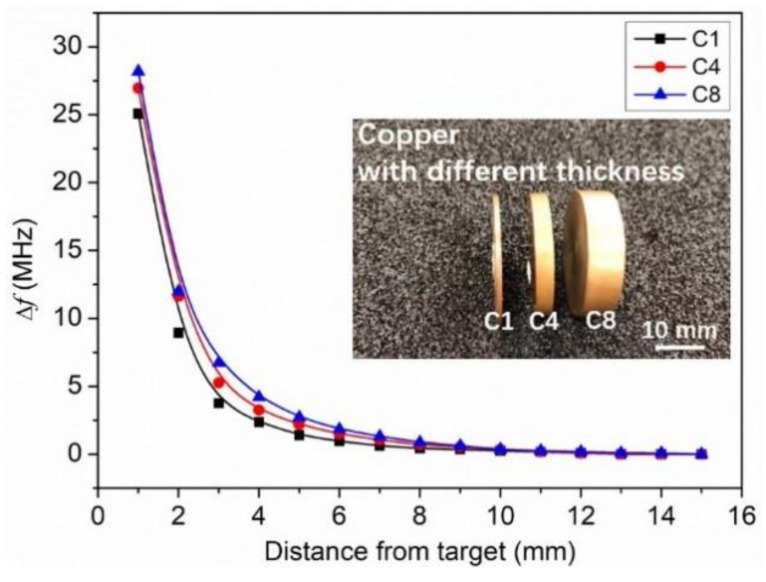
Frequency shift of the LC proximity sensor in dependence of distance from the targets of C1, C4, and C8.

The effect of the thickness of the metal target on the LC proximity sensor is also investigated. Three copper plates with the same surface area but different thicknesses of 1, 4, and 8 mm (namely C1, C4, and C8) were tested. Figure 8 shows the frequency shift of the LC proximity sensor in dependence of distance from the targets of C1, C4 and C8. A decrease in Δ*f* with the decreases of the thickness has been observed. This could be attributed to the influence of metal thickness on the eddy current. The thinner the thickness is, the smaller the eddy current is, and therefore the lower the sensitivity [23].

## 6. Conclusions

A passive wireless LC proximity sensor based on the LTCC technology was introduced. The wireless sensing mechanism of the LC proximity sensor to the metal targets was comprehensively investigated. Two types of target materials (copper and aluminum) have been studied. A higher sensitivity was observed in the copper target, which resulted from its lower resistivity value. The proximity sensing experiments using targets with different diameters and thickness showed that the frequency shift of the LC proximity sensor decreases with the reduction of the surface area and thickness of the metal target. The LC proximity sensor demonstrated a high sensitivity of 11.2 MHz/mm in the distance range of 1–3 mm, and a long detection distance up to 10 mm for the copper target. The good sensitivity and long detection range demonstrated that it could be a promising proximity sensor for wireless metal detections and distance measurements.

## Figures and Tables

**Table 1:** Parameters of the designed LC proximity sensor.

Symbol	Definition	Value
*d*_o_	Outermost of coil width	20 mm
*d*_i_	Innermost of coil width	11.5 mm
*w*	coil wire width	0.5 mm
*s*	the distance between two wires	0.25 mm
*N*_i_	number of turns of coil	6
*l*_c_	length of the fingers	8.5 mm
*N*_c_	number of fingers on each terminal	16
*d*_s_	spacing between two adjacent fingers	0.1 mm
*d*_c_	total spacing between two adjacent fingers	0.5 mm

**Table 2:** Details of the metal targets used in this work.

Metal Target	Copper						Aluminum
Sample code	C80	C45	C15	C8	C4	C1	A80
Diameter (mm)	80	45	15	25	25	25	80
Thickness (mm)	5	5	5	8	4	1	5
Resistivity (×10^−8^ Ω⋅m)	1.7						2.6
Permeability (×10^−6^ H/m)	1.25						1.25
